# Resident and Family Engagement in Care Conferences: Important Processes and Supporting Strategies

**DOI:** 10.1093/geroni/igab046.1040

**Published:** 2021-12-17

**Authors:** Tonya Roberts, Elizabeth Cox, Thuy Dan Tran, Hannah Tepsa

**Affiliations:** 1 University of Wisconsin - Madison, Madison, Wisconsin, United States; 2 University of Wisconsin, Madison, Wisconsin, United States

## Abstract

Self-determination is a core value of person-centered care. Research has shown residents and families want to be involved in decisions about care. Care conferences are one existing structure where residents and families can engage in decision-making about care goals. However, there are few tools to support effective engagement. To inform future tool development, this study sought to understand what resident and family stakeholders value about engaging in care conferences. In virtual meetings, 16 stakeholders identified 3 key areas of engagement: being informed about health/well-being, influencing care goals, and advocating for needs. They indicated current approaches do not achieve these engagement goals, which is particularly problematic during COVID when families cannot engage in person. Stakeholders offered ideas for supporting engagement such as provision of data before the conference. The study has implications for individualizing care conferences and encouraging resident and family engagement in decision-making both during and beyond COVID.

